# Design and application of an AI- and AR-enhanced serious game for interactive learning in the Blue Calico Museum in China

**DOI:** 10.1038/s41598-026-45304-8

**Published:** 2026-04-11

**Authors:** Yulin Yan, Jiajia Zhao, Euitay Jung

**Affiliations:** 1 Major of Visual Design, Department of Design, Graduate School, Hanyang University, Seoul, 04763 Republic of Korea; 2 Department of Communication Design, College of Design, Hanyang University ERICA, Ansan, 15588 Republic of Korea

**Keywords:** Artificial intelligence (AI), Augmented reality (AR), Serious games, Blue Calico, Cultural heritage museum, Cultural and media studies, Cultural and media studies, Information systems and information technology, Science, technology and society

## Abstract

With the widespread application of Artificial Intelligence (AI) and Augmented Reality (AR) technologies in the field of cultural education, technology-integrated serious games are increasingly emerging as effective tools for the dissemination of intangible cultural heritage. This study targeted university students and developed and deployed an AI- and AR-based serious game titled Dye Verse at the China Blue Calico Museum, serving as a museum-based learning intervention for higher education audiences. The game integrates features such as character creation, semantic guidance, AR recognition, and navigation, aiming to enhance students’ immersion and learning motivation. An experimental study involving 60 participants (N = 60) was conducted using pre- and post-knowledge tests and the User Experience Questionnaire (UEQ). Statistical analysis indicates that the experimental group significantly outperformed the control group in dimensions such as cultural knowledge acquisition, interactive engagement, and emotional identification. These findings validate the educational potential and communicative advantages of integrating AI and AR technologies in serious games. The study offers a cost-effective and highly interactive digital solution for small and medium-sized museums and provides both theoretical and practical implications for the sustainable dissemination of cultural heritage through gamified education.

## Introduction

With the advancement of AI and AR technologies, these tools have been increasingly applied in the field of education. Due to their immersive and practical characteristics, they are considered effective in enhancing learner engagement and improving educational outcomes^1^. In museum settings, AI and AR can enrich visitors’ experiences and deepen their understanding of cultural heritage^2^. Since being listed as one of the first National Intangible Cultural Heritage items in 2006, Nantong Blue Calico has gradually evolved from a utilitarian textile into a symbol of Chinese cultural and artistic spirit^3^. Enhancing user experience at the Nantong Blue Calico Museum through AI and AR technologies can significantly increase visitor participation and improve the retention of cultural content.

In the field of serious game design, extended reality (XR) systems incorporating AI and AR technologies have been shown to improve engagement and effectiveness in cultural heritage learning, offering new directions for the future development of virtual museums and gamified education^4^. For example, at the Natural History Museum of Funchal in Portugal, the AR-based game Memories of Carvalhal’s Palace—Haunted Encounters centres on a ghost-themed mystery, using AR technology and gamification to enhance both the entertainment value and educational outcomes of the museum experience. The project received positive responses, especially among younger audiences^5^. In the British Museum’s exhibition on Qajar-era Iranian playing cards, although no AR technology was used, Morris (2024) highlighted the issue of “sensory isolation” in museum displays, offering valuable insights for designing interactive experiences using AI and AR^6^. A study conducted at the National Museum of Natural Sciences (MNCN) in Madrid, Spain, demonstrated that collaborative learning in the AR game Enigma Ciencia MNCN was more effective than individual learning, enhancing both educational outcomes and the overall visitor experience. This approach helped break the limitations of traditional static displays and transformed the museum into a more participatory and interactive learning space^7^. At the Caracol Science Museum in Mexico, Cordova-Rangel and Caro (2023) designed and evaluated Aventura Marina, a serious game integrating escape room mechanics. This project showcased how highly interactive game structures can be combined with museum content to enhance visitor engagement and learning outcomes^8^. At the Emperor Qinshihuang’s Mausoleum Site Museum in China, a VR-based serious game centred on the Terracotta Warriors was developed to overcome the difficulty of close-range interaction, effectively enhancing public engagement and the dissemination of cultural heritage^9^. These cases offer valuable practical insights for the serious game design of the Blue Calico Museum in China, underscoring how AI- and AR-driven serious games can not only provide visitors with richer and more interactive experiences but also significantly enhance the communication of cultural heritage—ultimately achieving a meaningful fusion of education and entertainment.

Many serious games in museums currently face several challenges, including a mismatch between game content and users’ cognitive levels, inconsistent difficulty in instructional design, high technical costs and equipment requirements, as well as the complex balance between digital and physical experiences^10^. Therefore, the design of serious games must comprehensively consider how to implement challenges of varying difficulty levels and how to enhance visitors’ learning outcomes and exhibit retention through multisensory interactions.

To address the challenges faced by serious games in museums, this study proposes a solution based on the integration of AI and AR technologies. By leveraging AI to analyse visitor group profiles and generate narrative content, the attractiveness of exhibits can be significantly enhanced. Meanwhile, the application of mobile AR enables the combination of smart devices and interactive interfaces. Together, AI and AR technologies offer museums more personalized and immersive experience models, thereby improving the dissemination of cultural heritage and promoting innovation in museum-based education.

The findings of this study provide strong support for enhancing visitor interaction, cultural heritage education, and dissemination effectiveness in museums. By offering personalized gaming experiences through AI technology and leveraging mobile-based AR to circumvent the issue of limited financial resources, the proposed solution addresses existing challenges in the Blue Calico Museum of China—particularly the lack of interactivity and the imbalance between educational and entertainment functions. This innovative serious game design ultimately contributes to improving the overall museum experience.

In addition, the learning process facilitated by AI-driven interaction in serious games can be interpreted through the lens of Self-Regulated Learning (SRL). SRL emphasizes learners’ ability to actively plan, monitor, and evaluate their learning process^11^. In the context of museum-based serious games, AI-guided dialogue and task-based exploration can support learners’ goal setting, learning monitoring, and reflective engagement, thereby promoting deeper cultural understanding.

This study aims to address the following key research questions:

- *Q1*: How can AI and AR technologies be utilized to enhance the interactivity and learning effectiveness of serious games in museums?
- *Q2*: How can a balance be achieved between educational value and gameplay in museum-based serious games?
- *Q3*: How can the sustainability and long-term dissemination of cultural heritage be ensured in the design of serious games?

## Literature review

### Cultural value of Blue Calico and the need for digital preservation

The origin of blue calico can be traced back to the late Ming and early Qing dynasties, with the Nantong region emerging as a prominent production centre. During this period, large-scale cultivation of indigo plants began in Nantong, providing an abundant supply of raw materials for blue calico production. The dyeing techniques were also continuously improved, particularly through the adoption of the “paper-scraping resist dyeing” method, which enhanced the stability of the dyeing process and the vibrancy of the colours. Over time, Nantong blue calico evolved into a traditional craft characterized by a strong regional identity^12^.

Blue calico is renowned for its cobalt blue coloration, vibrant patterns, auspicious symbolism, and exquisite dyeing craftsmanship. It has been officially listed as one of the first entries in China’s National Intangible Cultural Heritage catalogue. However, with the evolution of societal aesthetics, the rustic colour tones of blue calico no longer align with modern visual preferences. In addition, its limited functional applications have led to a gradual decline of blue calico products in the consumer market^13^. Therefore, beyond preserving its traditional techniques and artistic value, it is crucial to explore innovative applications and cultural regeneration strategies for blue calico in contemporary contexts.

Liu and Ruangchewin have explored the use of laser engraving and digital design techniques to enhance both the production efficiency and aesthetic expression of blue calico, thereby achieving innovative development of traditional craftsmanship and promoting its sustainable cultural transmission^14^. In addition, Ye et al. examined the relationship between Nantong blue calico and the art of paper cutting. Their study suggests that paper-cutting not only influenced the pattern design of blue calico but also contributed to the evolution of its production techniques. The two art forms share notable similarities in terms of expressive style, cultural symbolism, and methods of transmission^15^.

To support the digital preservation of blue calico, Jia and Liu proposed an algorithm for extracting individual element sub-images from blue calico patterns. They further developed a classification method based on an improved CifarNet convolutional neural network architecture, named Calico Net, which successfully enabled automatic classification of design elements^16^. In a related effort, Yu et al. constructed and publicly released the NtBC dataset, which contains 43,209 blue calico pattern images across four themes and 34 categories. This comprehensive dataset provides a valuable resource for the digital preservation and cultural transmission of Nantong blue calico^17^.

In the area of image description, Guo et al. introduced the SLGCAN model and SLGCAM module, offering an innovative approach to generating image captions for blue calico patterns. Their method not only improved the accuracy of image descriptions but also provided new technical support for the digital preservation and presentation of blue calico heritage^18^. In addition, Sun et al. applied deep learning models to the intelligent classification and recognition of blue calico patterns. They constructed a comprehensive database of Nantong blue calico designs and employed the SSD (Single Shot MultiBox Detector) model with optimized adjustments, significantly improving the recognition accuracy of pattern identification^19^.

These studies indicate that the preservation of blue calico must evolve with the times by actively integrating modern technologies and digital dissemination strategies. Striking a balance between cultural inheritance and innovation is essential. Through the application of digital technologies, this traditional craft can be effectively preserved and promoted, allowing it to gain renewed vitality and relevance in contemporary society.

### Application of serious games in museums

Serious games refer to games that, beyond entertainment, are designed to achieve specific goals—often educational or training-oriented—through active player participation. These games have been widely adopted in the field of education and have also expanded into sectors such as healthcare, military training, business, and social work^20^. A meta-analysis conducted by Wouters et al. demonstrated that serious games outperform traditional instructional methods in terms of both learning outcomes and knowledge retention, providing empirical support for the effectiveness of serious games in promoting meaningful learning^21^. In the context of museums, the gamified approach to exhibition viewing has increasingly gained attention and support from visitors. By integrating elements of play, museums can offer more engaging and informal learning experiences, making cultural content more accessible and memorable^22^.

However, in many museums, the interaction between exhibits and visitors remains largely unidirectional, lacking sufficient interactivity. To address this issue, serious games have been introduced into museum environments to situate artifacts within specific narrative contexts, allowing visitors to experience the historical background and original functions of the objects—without compromising the integrity of the artifacts themselves^23^.As a result, cultural institutions have increasingly adopted serious games in recent years as tools for promoting both cultural and creative industries^24^. For instance, Coenen et al. designed MuseUs, a smartphone-based pervasive serious game that enables personalized museum experiences by eliminating the need for predefined tour routes and minimizing disruption to visitors’ natural behaviour. They also proposed a high-level architecture for designing a cultural heritage–oriented serious games^25^. Similarly, Türkmen and Savasta proposed the use of role-playing game (RPG)–based serious games in the İzmir Archaeology Museum to enhance visitors’ understanding and perception of cultural heritage through immersive gameplay^26^.In another example, Li et al. focused on Chinese bronze artifacts, using 3D laser scanning to create virtual models and developing a VR-based museum curation game. Their approach offers new possibilities for preserving and interpreting valuable cultural heritage through gamified digital exhibitions^27^.

The design of serious games in museums must go beyond mere entertainment and place equal emphasis on educational value. Beavis et al. demonstrated how to achieve a balance between educational content, authenticity, and engagement by examining the perspectives and priorities of three different stakeholder groups^28^. To further support this balancing act, Martinez et al. developed the Gaming Educational Balanced (GEB) model, which provides a structured framework for evaluating and designing serious games that harmonize entertainment and educational objectives. This model offers valuable guidance for the development of serious games in museum settings^29^.

Moreover, to prevent serious games from becoming conventional games with superficial educational overlays, it is essential that they be co-developed by interdisciplinary teams. These teams typically include educators, art directors, game designers, software developers, as well as graphic and sound designers^30^. Collectively, these cases demonstrate the wide-ranging applications of serious games in museums, particularly in enhancing interactivity, educational value, and overall visitor experience. The reviewed studies offer both theoretical foundations and practical references for the design and implementation of serious games in cultural institutions, helping museums strike a meaningful balance between educational objectives and engaging entertainment.

In summary, the application of serious games in museums not only helps to overcome the limitations of traditional one-way exhibition formats but also offers a novel experiential model that integrates both entertainment and education. However, careful consideration must be given to maintaining a balanced relationship between these two aspects during the design and implementation process.

### Application of AI technology in museums

In recent years, with the rapid advancement of Artificial Intelligence (AI), its application in museums has become increasingly widespread^31^. AI systems are capable of developing highly adaptive and intelligent personalization strategies that enhance the overall user experience in museum environments^32^.For example, Varitimiadis et al. investigated the use of AI chatbots in museums and applied the Analytic Hierarchy Process (AHP) to evaluate different conversational platforms and chatbot systems. Their findings provide museums with practical guidance for selecting the most appropriate dialog-based AI solutions^33^. In another case, Liang et al. addressed the limitations of traditional museum education in providing learning support by incorporating AI chatbots into Alternate Reality Games (ARGs). These AI agents offered personalized assistance to learners lacking prior knowledge or metacognitive skills, significantly improving their ability to complete tasks within the game-based learning environment^34^.

To address the challenges of dull information presentation and limited interactivity in traditional museums, Wen and Ma developed an adaptive Convolutional Neural Network (CNN) model for the automatic identification and classification of exhibit images. Their system enables visitors to explore exhibit details and related narrative backgrounds through gesture recognition, touchscreen interaction, and voice commands, thereby enhancing user engagement and immersive learning^35^. In addition, AI has played a significant role in the field of artifact restoration. Recent research has combined Stable Diffusion (SD) and Neural Radiance Fields (NeRF) models to restore damaged cultural heritage objects and generate highly realistic 3D digital surrogates. This approach substantially improves the accuracy and fidelity of digital archiving for museum collections^36^.

While AI is being widely adopted in museum contexts, its use has also raised growing ethical concerns. For instance, Fu et al. emphasized that when implementing AI technologies for digital heritage display, museums must collaborate closely with cultural experts to ensure "augmented authenticity." This approach helps prevent cultural misinterpretation and distortion, while also enhancing audience comprehension and engagement^37^.

These cases highlight the advantages of applying AI technologies in museums, particularly in enhancing interactivity, enabling personalized learning experiences, supporting intelligent exhibit narration, and facilitating artifact restoration. They provide important theoretical support and practical direction for the digital transformation and intelligent development of museums. Moreover, these insights offer valuable reference points for the design and implementation of the present study.

### Application of augmented reality (AR) technology in museums

With the advancement of smart devices, sensors, software development kits, and network technologies, Augmented Reality (AR) has demonstrated significant potential across various industries, including cultural tourism, entertainment, manufacturing, and education. As core institutions for both culture and learning, museums have actively explored the integration of emerging technologies into visitor education strategies. AR-based applications in museums overlay virtual content onto physical environments, offering visitors a more immersive and multisensory experience^38^.

The application of AR technology has introduced multiple innovative approaches to enhancing the visitor experience in museums. AR enables the delivery of contextual information that extends beyond the physical artifacts or historical sites themselves, allowing for more interactive and meaningful forms of communication with cultural content^39^. As the adoption of AR in museum contexts continues to expand, a growing body of research has begun to explore its potential and practical effects within cultural heritage environments.

For example, Bekas and Xinogalos developed the serious game Exploring Ancient Greece, which uses AR technology to present archaeological sites from ancient Greece. Their study highlights the significant potential of AR in the educational domain, particularly in enhancing students’ engagement with historical learning. The game also offers valuable insights for museums seeking digital transformation strategies^40^. In another case, Tokuno et al. designed an AR–AR collaborative game specifically for children visiting museums. The game incorporated a “searchlight” user interface and utilized tilt-based controls to enable joint actions between players. This design enhanced the interactivity of the museum experience for children and demonstrated the advantages of AR in promoting social engagement and collaborative learning in younger audiences^41^.

In addition, He et al. conducted an in-depth study on the impact of AR design elements on museum tourism experiences. Their findings suggest that virtual environments can function as contextual cues that facilitate psychological responses and enhance visitors’ aesthetic experiences. The study emphasizes that museums can significantly improve user experience through the thoughtful integration of localized virtual features, without requiring large-scale or high-cost technological investments^42^.

Tang and Zhou, through a series of six experimental studies, found that AR technology significantly enhances visitor satisfaction in historical museums by helping them establish intertemporal connections between the past, present, and future. Their work offers valuable insights into how museums can effectively deploy AR to enrich visitor experiences^43^. Yi and Chen proposed an integrated design methodology combining Kano, AHP, and QFD models to systematically guide the development of AR applications for cultural heritage museums based on user needs. This framework provides scientific support for user-centred AR design^44^. Hu et al. incorporated both AR and blockchain technologies in the interactive design of digital museums. By conducting comparative experiments in two museums in Beijing, they demonstrated practical strategies for digital transformation within cultural institutions^45^. These cases collectively illustrate the diverse benefits of AR technology in museum education—improving interactivity, user experience, learning outcomes, and social engagement. They also provide both theoretical and practical guidance for this study’s exploration of AR-enhanced serious games in cultural heritage contexts.

In summary, the application of AR technology in museums offers immersive and highly interactive experiences, making it a key driver in the digital transformation of museums, cultural dissemination, and educational innovation. Through its practical implementation in this study at the Blue Calico Museum in China, the adaptability and effectiveness of AR technology in the dissemination of intangible cultural heritage were further validated. The AR-enhanced approach not only improved visitors’ cultural understanding and engagement but also provided small and medium-sized museums with a cost-effective and highly interactive exhibition model.

## Methodology and framework

**Fig. 1:**
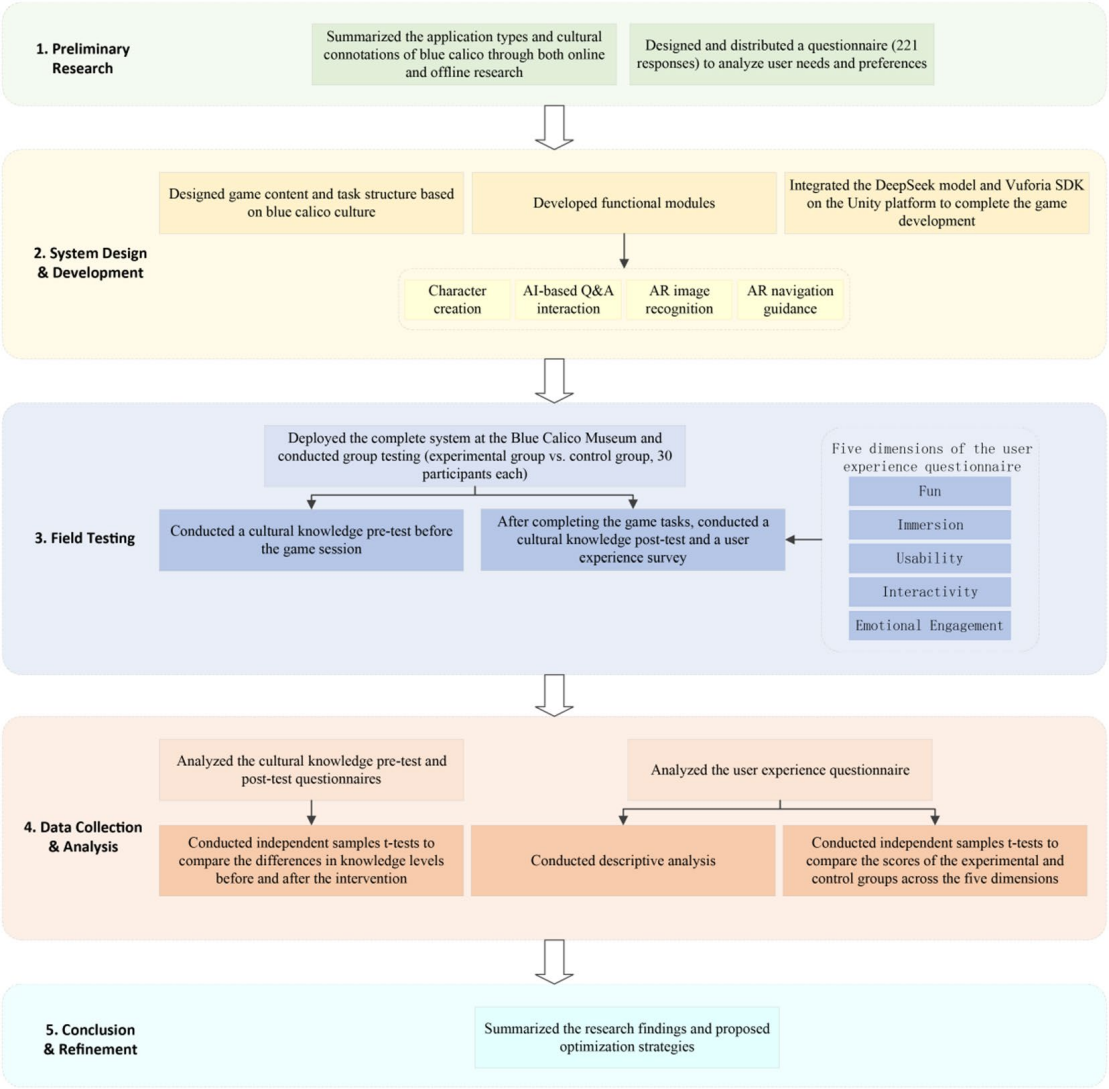
Research process.

This study adopts a combination of design-based research (DBR) and quasi-experimental methods to explore the application of AI and AR technologies in the context of intangible cultural heritage dissemination at the Blue Calico Museum in China. A serious game system integrating AI-driven interaction and AR-based navigation was developed and evaluated through field testing and user feedback. The study aims to assess the system’s effectiveness in enhancing cultural awareness, user engagement, and immersive experience. The research process was divided into four stages: the preliminary research stage, the system development stage, the field testing stage, and the data analysis stage, as illustrated in Fig. 1.

During the preliminary research stage, core cultural elements of Blue Calico were extracted through literature analysis, field observation, and in-depth interviews. In addition, public preferences regarding museum visiting experiences and digital communication formats were investigated. Based on the findings of this stage, the thematic composition, interaction logic, and educational objectives of the serious game were determined, providing both cultural and functional foundations for the system design.

During the system development stage, this study utilized AI and AR technologies to construct a serious game system themed around Blue Calico. The system comprises three core modules: an AI-based question-and-answer module, an AR pattern recognition module, and a contextualized learning task module. Through multiple rounds of testing and optimization, a functional prototype system with strong stability and engaging interactivity was developed.

During the field-testing stage, the game system was deployed at the China Blue Calico Museum, where 60 local university students were invited to participate in interactive gameplay. The participants were divided into an experimental group and a control group: the experimental group used the complete AI + AR serious game system, while the control group used a basic version without intelligent functions. All participants experienced the game tasks under unified guidance and subsequently completed follow-up questionnaires and tests.

Data analysis focused on questionnaire surveys, knowledge tests, and interview feedback to comprehensively evaluate the system’s practical effectiveness in intangible cultural heritage communication and user experience.

The specific components include:

1. Knowledge pre-test and post-test: used to measure changes in participants’ understanding of Blue Calico cultural knowledge before and after the game intervention;
2. User experience questionnaire: evaluating participants’ subjective experiences across five dimensions—fun, immersion, usability, interactivity, and emotional engagement;
3. Interview analysis: collecting open-ended feedback to understand users’ overall impressions of cultural learning and interactive experience.

In terms of statistical methods, SPSS software was used for data processing, primarily employing descriptive statistical analysis (mean and standard deviation calculations) and independent-sample t-tests to compare differences and significance levels between the experimental and control groups across various indicators. This analysis aimed to verify the practical effectiveness of the AI + AR serious game in the dissemination of intangible cultural heritage. (Table 1)

**Table 1:** Cultural elements of Blue Calico.

Cultural characteristics	Description
Pattern and motif design	Blue calico is generally categorized into two types: white patterns on a blue background and blue patterns on a white background. The pattern design emphasizes simplicity and harmony, often drawing inspiration from natural imagery. The motifs typically combine dots, lines, and planes, resulting in visually rich and structured compositions. The pattern types are diverse, including Chinese characters, plants, animals, human figures, symbols, and geometric shapes. Each pattern carries its own cultural symbolism and aesthetic value, reflecting traditional beliefs, ethical ideals, and regional identity embedded in everyday visual culture
Production techniques	The production process of blue calico is relatively complex and involves nine primary steps: preparing the base fabric, degumming, stencil carving, applying tung oil, paste scraping, dye preparation, dyeing, paste removal and rinsing/drying, and post-finishing. The dyeing process uses natural indigo extracted from indigofera plants and relies on natural fibers such as cotton or linen as the base material. All procedures are conducted entirely by hand, following traditional techniques. The fabric is repeatedly immersed in the dye solution to achieve high colour fastness and a deep, vibrant hue. This manual, multi-step method ensures not only the durability and aesthetic quality of the patterns but also reflects an environmentally friendly and sustainable approach to textile production
Regional folk culture	Blue calico originated in Nantong, a region whose favorable geographical conditions and stable social environment contributed to the development of this traditional craft. The introduction of Polygonum tinctorium (indigo plant) cultivation techniques by merchants from Guangdong further supported the establishment of a robust dyeing industry. Over time, blue calico became a representative traditional craft of the Jiangnan region, embodying both local cultural identity and artisanal heritage
Ecological symbolism	The indigo dye used in blue calico is derived from natural plants, specifically Polygonum tinctorium (Japanese indigo), making it an eco-friendly, non-toxic, and harmless natural dye. Its use aligns well with contemporary principles of sustainable development, highlighting the environmental consciousness embedded in traditional textile practices
Auspicious meaning and symbolism	The patterns in blue calico are rich in auspicious symbolism, conveying traditional Chinese values and hopes for a better life. For example, “pine and crane for longevity” (松鹤延年) symbolizes long life; “carp leaping over the dragon gate” (鲤鱼跳龙门) represents success and upward mobility; and “lion playing with embroidered ball” (狮子滚绣球) reflects wishes for joy, prosperity, and family harmony. The use of color also carries symbolic meaning: blue represents calmness and depth, while white symbolizes purity and sacredness. These symbolic associations enhance the emotional and cultural resonance of blue calico, making it more than just a textile—it is a medium of visual storytelling and spiritual expression

## System design and practical implementation

### Preliminary research

**Fig. 2:**
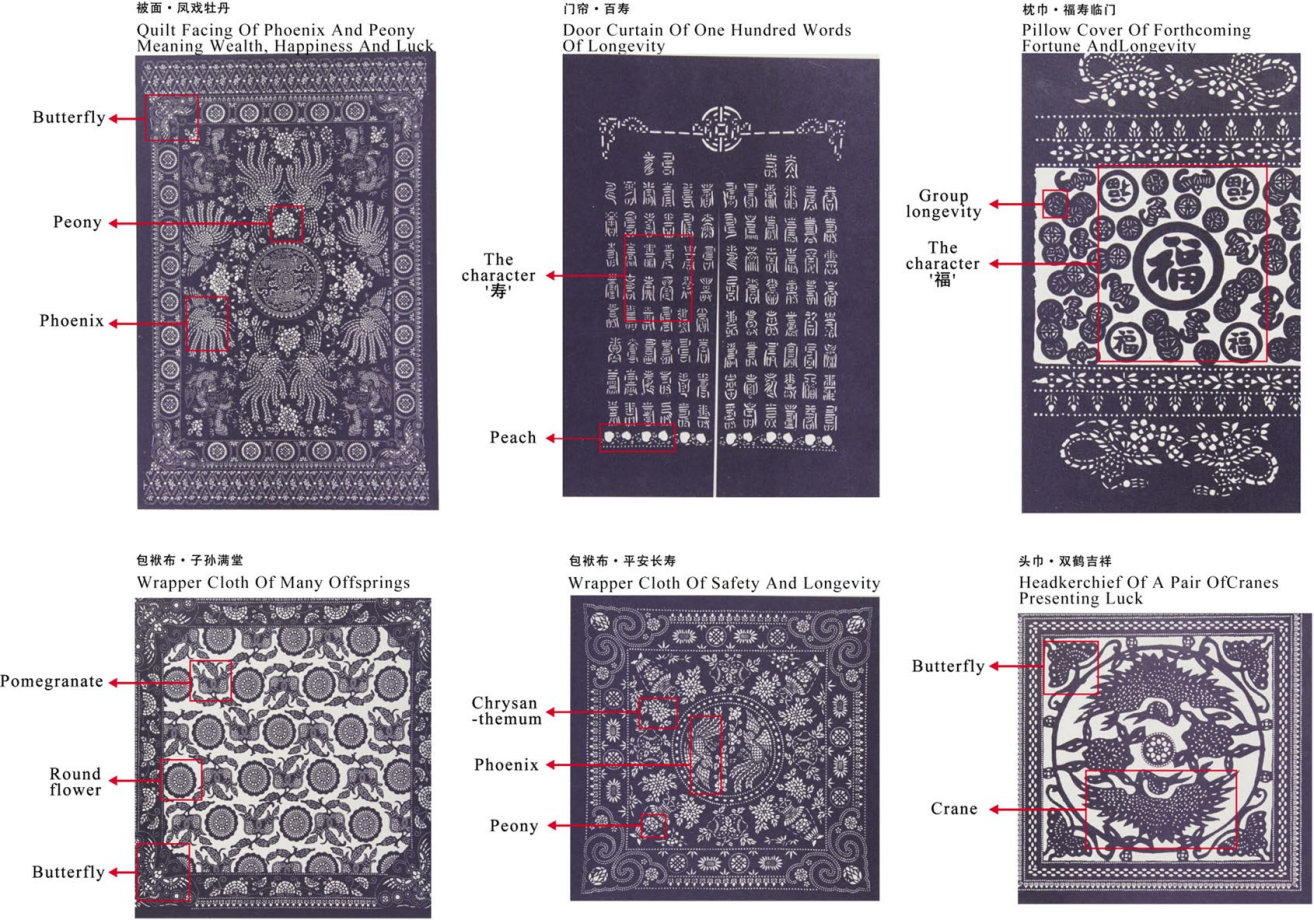
Common pattern motifs in Blue Calico.

The preliminary research of this study was conducted based on the Nantong Blue Calico Museum. Through a combination of on-site and online investigations, along with an in-depth analysis of the book An Overall Collection of China Blue Calico Vein Patterns compiled by Wu Yuanxin^46^, a recognized inheritor of the Nantong blue calico tradition, the types and cultural connotations of blue calico were systematically summarized. Common application types include quilt covers, door curtains, pillow towels, wrapping cloths, and headscarves. Frequently used design elements involve peonies, phoenixes, Chinese characters, longevity symbols, and chrysanthemums. Representative patterns are shown in Fig. 2. The key cultural elements of blue calico encompass pattern design, dyeing and printing techniques, local cultural references, ecological symbolism, and auspicious meanings. These dimensions are detailed in Table 2. Together, they constitute the deep cultural significance of blue calico and provide a foundational reference for the subsequent stages of serious game design.

**Table 2:** Summary of sample characteristics (n = 221).

		Frequency	Percentage (%)
Age	18 and below	9	4.10
	19–30 years old	144	65.20
	31–40 years old	47	21.30
	41–50 years old	20	9.00
	51 and above	1	0.50
Region	Within Jiangsu Province	35	15.84
	Outside Jiangsu Province	186	84.16

As part of the preliminary research phase, a questionnaire survey was conducted to gather feedback on participants’ museum experiences and preferences. A total of 221 valid responses were collected and analysed using SPSS. The sample distribution covered variables such as gender, age, occupation, and frequency of museum visits. The analysis revealed that most respondents were between 19 and 30 years old, with a large proportion being students. Regarding museum visit frequency, most participants reported visiting museums annually or quarterly, while a smaller group indicated that they visit monthly. Overall, the sample showed a good degree of representativeness in terms of demographic and behavioural characteristics. Detailed demographic statistics are presented in Table 2.

In addition, to ensure the reliability and validity of the research conclusions, the SPSS software was used to assess the internal consistency of the questionnaire. The Cronbach’s Alpha coefficient was calculated to be 0.702, which meets the conventional threshold for acceptable reliability. This indicates that the questionnaire is capable of effectively reflecting the relevant dimensions of the research objectives.

Based on the results of the nine questionnaire items, a clear correlation was observed between visitors’ willingness to use AR and VR technologies in museums and their prior experience with such technologies. Visitors who had previously used AR or VR expressed a significantly stronger desire to incorporate these technologies into future museum experiences. Furthermore, a strong association was found between the perceived educational function of museums and the demand for interactive experiences, suggesting that enhancing both educational content and interactivity can substantially improve visitor satisfaction. In addition, visitors’ interest in exhibits was closely related to their preference for interactive experiences, indicating that optimizing interactive exhibit design can effectively increase visitor engagement. Through this preliminary research, key cultural features and content themes of blue calico were identified and aligned with public expectations. These findings provided a critical reference for the subsequent design of the serious game system.

### Design objectives and core mechanisms of the serious game

With the support of AI and AR technologies, this study designed an educational yet entertaining serious game titled *“DyeVerse”*, aiming to create an immersive museum interaction experience for visitors. Through gameplay, participants are encouraged to actively explore, understand, and perceive the cultural connotations of Blue Calico.

The game is designed with three core objectives: cultural knowledge acquisition, immersive participation, and sustainable communication. It aims to promote the learning and retention of intangible cultural heritage through interactive experiences. By combining task-driven mechanisms with contextualized narratives, visitors can systematically understand the historical origins, production techniques, and symbolic meanings of Blue Calico patterns, thereby achieving a deeper cognition and effective transmission of intangible cultural heritage within an engaging and gamified experience.

In the game design, the system integrates multiple interactive mechanisms to achieve diverse learning objectives. Through narrative guidance, players gradually learn about the cultural background of Blue Calico. The role-playing mechanism allows players to participate as “inheritors,” reinforcing their sense of cultural responsibility. The game stages are divided into three progressive levels—“First Encounter with Blue Calico,” “Creative Blue Calico,” and “The Secret Patterns of Blue Calico”—to help visitors systematically acquire cultural knowledge. The AR recognition function enables interaction with physical exhibits, allowing players to obtain cultural information through observation. The NPC ‘AI Master Wu’ Q&A module guides players toward deeper learning, while the AR navigation map assists them in completing tasks and enhances spatial immersion.

From a learning theory perspective, the interactive mechanisms of the system also reflect the core processes of Self-Regulated Learning (SRL). The task-driven game structure helps players establish learning goals at different stages, while the semantic guidance and real-time question-and-answer support provided by the AI character “Master Wu” assist players in monitoring their learning progress and resolving knowledge gaps during exploration. By continuously completing tasks, receiving feedback, and revisiting exhibits, players are able to reflect on and adjust their understanding of the cultural knowledge acquired, thereby forming an active learning cycle.

To ensure alignment between game mechanics and learning objectives, the game’s core mechanisms were constructed based on the LM-GM (Learning Mechanics–Game Mechanics) framework proposed by Arnab et al. (see Table 3).

**Table 3:** Mapping between learning mechanics and game mechanics.

Game mechanics	Learning mechanics	Implementation method
Story Cutscenes	Guided Learning	Introduce mission objectives and background story through dialogue with the NPC
Role-Playing	Empathy and Sense of Responsibility	Role-play as a blue calico inheritor to immerse in traditional craftsmanship and tasks
Level-Based Mechanism	Goal Decomposition	Complete tasks through three stages: "Introduction to Blue Calico," "Calico Creation," and "Calico Secrets."
AR Image Recognition	Observation and Recognition	Trigger exhibit explanations by scanning physical items with AR
Virtual NPC (AI) Interaction	Inquiry	Ask "AI Master Wu" questions to acquire more knowledge about blue calico
AR Navigation and Map Guidance	Spatial Exploration	Guide players to complete interactive tasks along a designated route in the physical exhibition space

### Gamified design of Blue Calico cultural knowledge

The game conveys the cultural knowledge of blue calico through narrative storytelling, interactive Q&A, and task-based guidance. Centred around the storyline titled “The Secret Manual of Blue Calico”, the game narrates how historically significant blue calico patterns—once scattered across time—have reappeared in various exhibition zones of the Nantong Blue Calico Museum. Players, acting as inheritors of intangible cultural heritage in the modern era, are tasked with gradually identifying these patterns within the museum. By doing so, they unlock the historical origins, symbolic meanings, and production techniques associated with each design. Through completing designated tasks, players ultimately reconstruct the fragmented contents of the “Secret Manual of Blue Calico”.

For instance, in the opening stage of the game, the NPC “Master Wu” introduces the origins of blue calico through a dialogue-based interaction, stating: “The history of blue calico can be traced back to the Song and Yuan dynasties, reaching its peak during the Ming and Qing periods.” This provides players with an initial understanding of its historical background. In subsequent tasks, players use AR scanning to identify patterns such as “Bats with Peaches” and “Entwined Lotus”, obtaining information about their folkloric meanings and application contexts. During the “Blue Calico Creation” phase, players simulate the entire production process—carving stencils, applying soybean paste, dyeing, and paste removal. In the “Secret Manual” puzzle task, players locate corresponding fragments through the AR navigation system and assemble them to uncover the knowledge and symbolism embedded in the patterns. Additionally, the virtual NPC “Master Wu” provides on-demand explanations about blue calico via a Q&A format. Players can actively ask questions such as “What does the bat pattern represent?” or “Why does soybean paste applied?”, and the system returns concise knowledge summaries with visual illustrations, further guiding users to explore related exhibits. This mechanism encourages players to engage with the museum through inquiry-based interaction, allowing them to build a network of knowledge surrounding blue calico culture.

In selecting intangible cultural heritage (ICH) elements, priority was given to motifs with prominent visual features and rich symbolic meanings to facilitate AR recognition and player observation. Traditional patterns such as bats, longevity medallions, and pomegranates were chosen as core expressive elements. These motifs not only embody profound cultural significance but are also well-suited for transformation into AR-recognizable objects and virtual assets. From a technical perspective, an AI-powered pattern generation system was integrated into the game. Players can input keywords—such as “blessing” or “festivity”—to generate corresponding blue calico patterns, which can then be applied to virtual handicrafts and garments, thereby enhancing opportunities for creative expression. Additionally, through AR recognition, players can scan physical objects in the museum environment, such as printing blocks and dye vats, to trigger demonstrations of the blue calico production process. This approach enables a deep integration of traditional craftsmanship with contextual interaction.

### System architecture and functional module overview

**Fig. 3:**
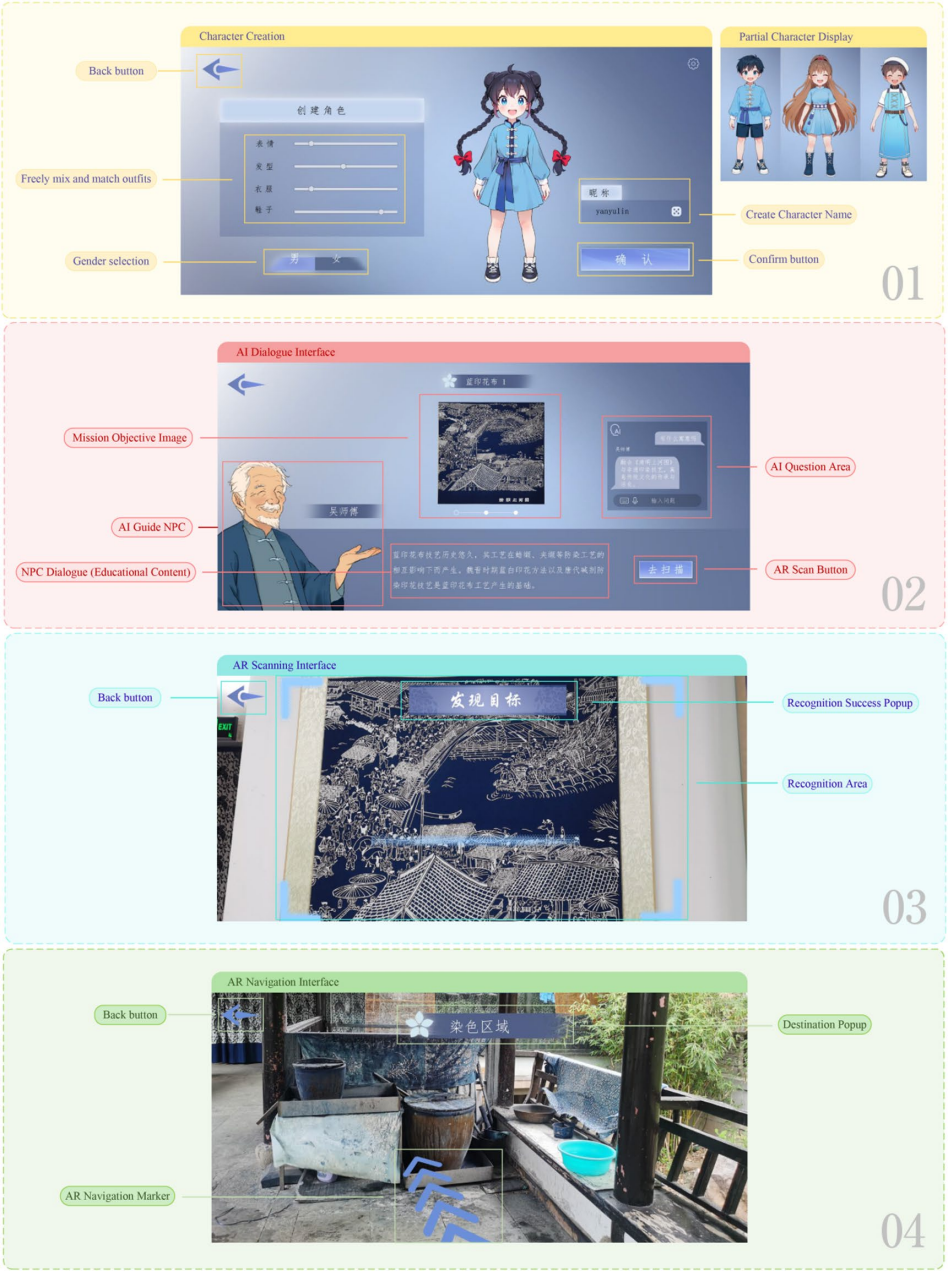
User interface of the serious game system.

This game is built upon the integration of AI and AR technologies, utilizing four interactive modes—recognition, dialogue, task execution, and navigation—to facilitate the dissemination of blue calico cultural knowledge. To ensure system stability and compatibility with mobile platforms, the entire platform is developed using Unity and integrated with the Vuforia SDK, supporting both Android and iOS systems. The system is composed of five core functional modules: Character Creation Module, AI Guidance Module, AR Recognition Module, Task Interaction Module, and Scene Navigation Module, as illustrated in Fig. 3.

In the character creation module, users can not only set the character’s name and select gender but also customize the character’s appearance and outfit through adjustable sliders, enabling a high degree of personalization. The costume design incorporates elements of blue calico, such as traditional Chinese frog buttons, to ensure visual consistency with the overall cultural theme.

**Fig. 4:**
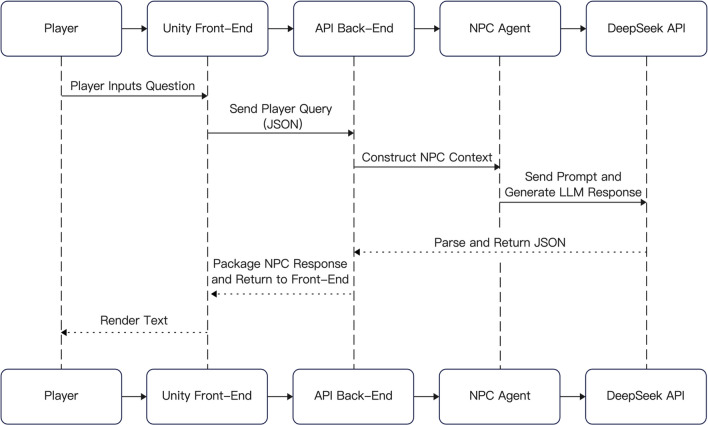
Data flow process diagram.

In addition, the system leverages AI technology to provide dynamic feedback during character interactions, adapting content presentation according to the player’s learning progress to strengthen the personalized learning experience. As noted by Tang and Liang et al., AI-driven personalized systems in narrative game environments can deliver tailored feedback based on players’ knowledge levels, thereby effectively enhancing learning outcomes^47^. In the present system, the AI-guided module functions as the core component for disseminating knowledge about blue calico, with the AI character “Master Wu” responsible for task explanation, cultural Q&A, and pattern interpretation. As shown in Fig. 4, this module integrates a DeepSeek interface powered by a large language model, enabling natural language queries and semantic comprehension. Within the AI dialogue interface, functional components such as a task objective map, AI query area, and navigation buttons are embedded. Players can actively engage in dialogue with the NPC by clicking on the question area. The system sends the input query via Unity through a RESTful API to the DeepSeek backend model, which generates a response that is then returned and displayed on the front-end interface in both text and voice formats. This interactive approach not only facilitates the transmission of cultural knowledge but also allows real-time adaptation based on the player’s task progress and contextual information, thereby enhancing the educational and interactive value of the serious game.

The AR recognition module of this system is primarily responsible for identifying exhibits. Players use the camera on their mobile device to scan blue calico patterns and related crafting tools within the museum. Upon successful recognition, a “Target Found” prompt is triggered, activating relevant content such as pattern interpretation, historical background, and interactive tasks. The design of this module draws upon the architectural framework of the GamIN platform for spatial cultural interaction^48^. The system integrates the Vuforia Engine SDK within the Unity platform, utilizing the Image Target feature for 2D image recognition, along with Ground Plane and Model Target functionalities for 3D anchor positioning and spatial content overlay. Prior to development, multiple site investigations were conducted to collect images of patterns, production tools, and exhibits for training recognition and constructing interactive content. During the development process, special attention was given to optimizing model assets for lower-end devices by compressing resources and limiting image recognition assets to under 1.5 MB, while ensuring a minimum three-star recognition rating. By associating physical exhibits with digital pattern markers, the system dynamically overlays visual and textual information onto real-world objects when a target is scanned. This enhances the visual expression and cultural engagement of blue calico through a layered digital-physical experience.

The AR navigation module is developed based on the spatial layout of the exhibition hall. Utilizing Unity in conjunction with Vuforia’s Ground Plane and Area Target technologies, the system projects virtual pathways and destination prompts within the real-world environment. This guides players from their current task location to the next designated task point. The integration of this feature enhances the continuity of the task flow and fosters a more immersive game experience as players navigate through the museum space.

### Practical deployment and exhibition strategy in the Blue Calico Museum

**Fig. 5:**
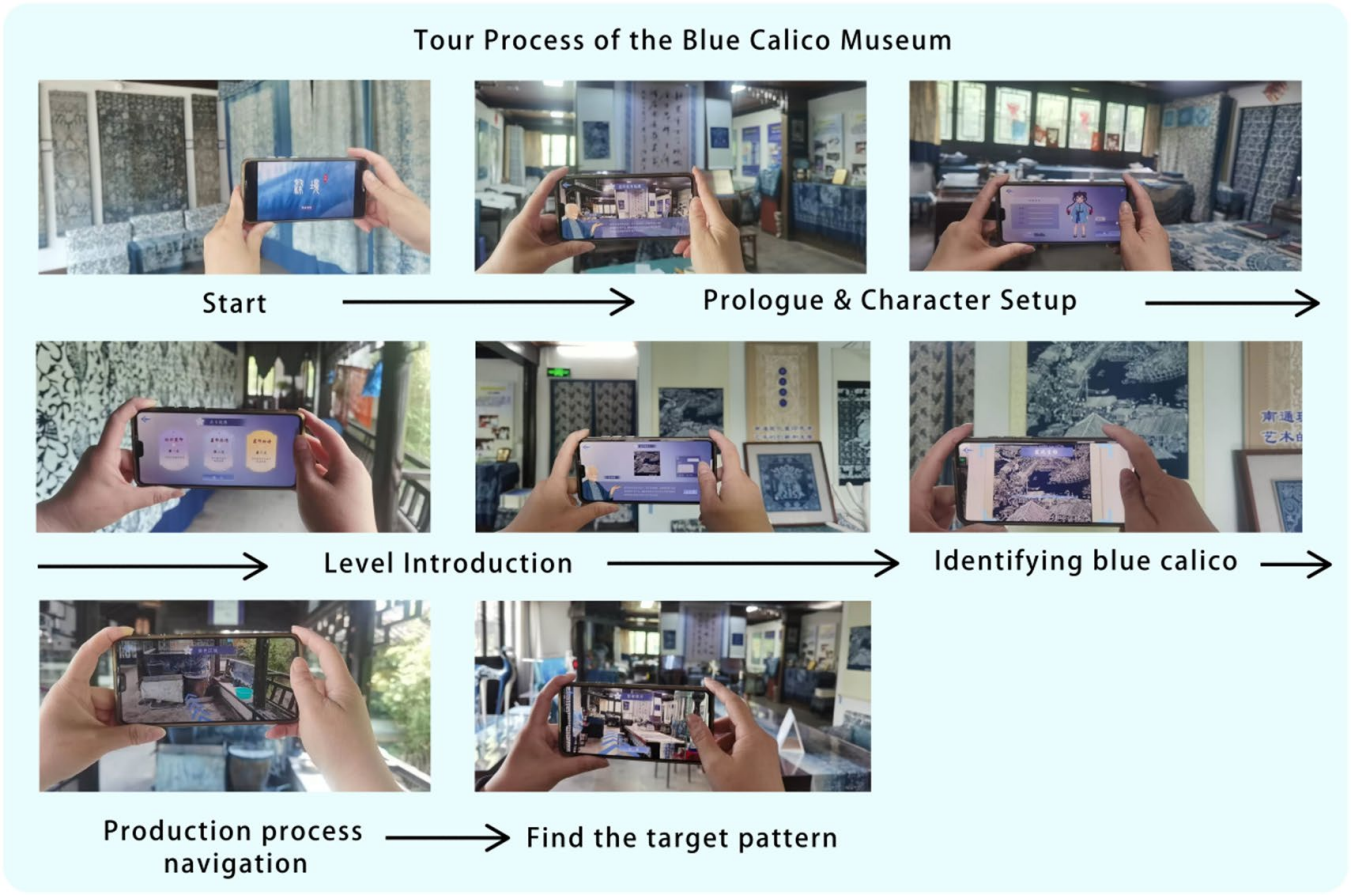
On-site deployment of the game system.

The game has been fully deployed at the Blue Calico Museum. At the museum entrance, players can scan a QR code to access the main interface of the game. Upon entry, users begin by creating a personalized character, selecting a nickname, gender, and clothing style, thus initiating a customized exploration journey. The game introduction is presented by the virtual NPC "Master Wu," who provides an overview of the origins of blue calico and the background narrative of the game. The gameplay is divided into three main stages: Pattern Recognition, Craftsmanship Experience, and Restoration of the Secret Calico Manuscript. Each task involves the collection of visual clues, triggering AR content, and receiving contextual interpretation from the NPC. Players are required to locate specific blue calico patterns or traditional tools and use their mobile device’s camera to perform AR scanning. Once an object is successfully recognized, the corresponding textual and visual information is presented. After completing each task, an arrow is projected onto the ground via the AR navigation system, guiding the player to the next exhibition zone. The journey continues through the carving area, dyeing station, and finished goods section. Additionally, the game includes a photo-recording feature, enabling users to capture interactive moments during gameplay. Upon completing the first two stages, players unlock the final task, completing the narrative loop of the game. The concrete deployment and interaction flow are illustrated in Fig. 5.

## Effectiveness evaluation and discussion

### Evaluation methods and assessment criteria

This study adopted two evaluation methods: a knowledge pre-test and post-test, and a user experience questionnaire.

(1) Pre- and Post-Knowledge Tests.

The same set of 10 multiple-choice questions related to blue calico culture was used for both the pre-test and post-test. The questions focused on key cultural content, including historical background, symbolic meanings of patterns, and the production process. These knowledge tests were administered before and after the game experience, aiming to measure changes in participants’ knowledge acquisition. The collected data were analysed using an independent samples t-test to determine whether there was a statistically significant difference in knowledge improvement between the experimental group and the control group.

(2) User Experience Questionnaire.

To evaluate participants’ perceptions and satisfaction during the serious game experience, this study adopted the short version of the User Experience Questionnaire (UEQ-S) developed by the European Human–Computer Interaction Group^49^. The assessment focused on five key dimensions: fun, immersion, ease of use, interactivity, and emotional involvement. The questionnaire consisted of 15 items, each rated on a five-point Likert scale (1 = strongly disagree, 5 = strongly agree). The collected data were analysed using SPSS to calculate mean values, standard deviations, and conduct significance testing to assess differences in user experience between groups.

### Participants’ feedback and data analysis results

#### Knowledge pre-test and post-test analysis

The cultural knowledge test consisted of 10 multiple-choice questions, covering key aspects of blue calico such as historical origins, symbolic pattern meanings, and production processes. Using SPSS, an independent samples t-test was conducted on the pre-test and post-test scores to compare the differences in knowledge levels between the experimental group and the control group before and after the intervention.

**Fig. 6:**
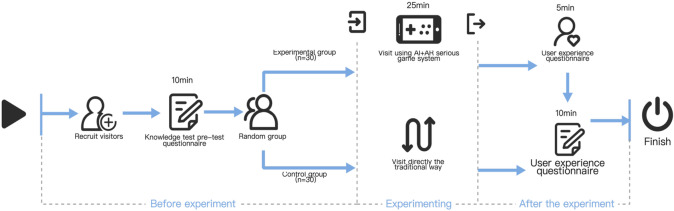
Experimental procedure.

The experiment was conducted in three stages: pre-experiment, during the experiment, and post-experiment. As shown in Fig. 6, a total of 60 undergraduate students were recruited before the experiment, with an average age of 22 years (20 males and 40 females). All participants voluntarily took part in the study and completed every stage of the experiment. They were randomly assigned to an experimental group (n = 30) and a control group (n = 30) and participated in both knowledge pre-tests and post-tests. During the experimental stage, the experimental group engaged in an immersive learning experience supported by AI + AR technologies, while the control group learned through traditional museum visits. After the experiment, the experimental group completed a user experience questionnaire, and both groups participated in the knowledge post-test.

Table 4 shows that the experimental group’s pre-test mean score was 4.20, while the control groups was 4.47. Although the control group scored slightly higher, the difference was minimal. The standard deviations were 1.270 and 1.358, respectively, indicating similar levels of dispersion. According to the Levene’s test for equality of variances in Table 5, F = 0.250, p = 0.619 > 0.05, suggesting no significant difference in variances, and thus the assumption of equal variances is met. Therefore, the "Equal variances assumed" row is used for the subsequent t-test. The independent samples t-test result shows t = -0.786, df = 58, p = 0.435, which is greater than 0.05. This indicates that the difference in pre-test total scores between the two groups is not statistically significant. The mean difference of -0.267 falls within the normal fluctuation range. Further effect size calculations showed that Cohen’s d = -0.205 and η^2^ = 0.0105, both of which are far below the conventional threshold for a small effect size. Hence, the two groups had a comparable baseline knowledge level prior to the intervention, providing a solid foundation for further experimental comparisons.

**Table 4:** Pre-test group statistics.

	Group	Mean	Std. Deviation
Pre-test Score	Experimental group	4.20	1.270
	Control group	4.47	1.358

**Table 5:** Pre-test independent samples test.

		Levene’s Test for Equality of Variances		t-test for Equality of Means				
		F	Sig	t	df	Sig. (2-tailed)	Cohen’s d	**η**^**2**^
Pre-test Score	Equal variances assumed	0.250	0.619	-0.786	58	0.435	-0.205	0.0105
	Equal variances not assumed			-0.786	57.745	0.435	-0.205	0.0105

**Fig. 7:**
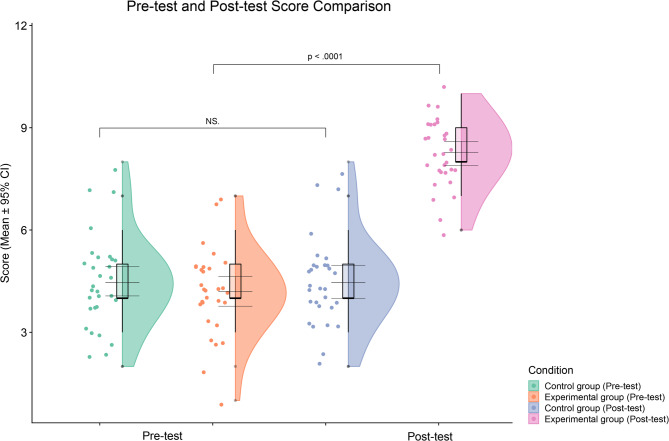
Distribution of pre- and post-test scores.

As shown in Table 6, the post-test mean score of the experimental group was 8.27 with a standard deviation of 1.048, while the control group had a mean score of 4.47 with a standard deviation of 1.358. The experimental group scored significantly higher than the control group, indicating a substantial improvement in knowledge acquisition. The result of the homogeneity of variance test for the post-test is presented in Table 7: F = 1.024, p = 0.316 > 0.05, indicating that the assumption of equal variances holds. Therefore, the result under the Equal variances assumed condition was adopted for subsequent analysis. According to the independent samples t-test, the result showed t = 12.133, df = 58, p < 0.001, suggesting a statistically significant difference in post-test scores between the two groups. In addition, the effect size indicators were Cohen’s d = 3.133 and η^2^ = 0.717, both indicating a very large intervention effect. As shown in Fig. 7, the detailed data distribution of the experimental and control groups in the pre-test and post-test stages demonstrates that the experimental group exhibited a significantly greater improvement in learning performance after the intervention compared to the control group.

**Table 6:** Post-test Group Statistics.

	Group	Mean	Std. deviation
Post-test score	Experimental group	8.27	1.048
	Control group	4.47	1.358

**Table 7:** Post-test independent samples test.

		Levene’s test for equality of variances		t-test for equality of means				
		F	Sig	t	df	Sig. (2-tailed)	Cohen’s d	η^2^
Post-test score	Equal variances assumed	1.024	0.316	12.133	58	0.000	3.133	0.717
	Equal variances not assumed			12.133	54.508	0.000	3.133	0.717

To further verify the significance of the intervention effect, this study calculated each participant’s improvement score, defined as the post-test score minus the pre-test score. An independent samples t-test was conducted to compare the improvement between the experimental and control groups. As shown in Table 8, the experimental group had an average improvement score of 4.07 with a standard deviation of 1.552, while the control group showed no change, with an average improvement score of 0 and a standard deviation of 0. This indicates that the control group experienced virtually no change between pre- and post-tests, whereas the experimental group demonstrated significant improvement. According to the Levene’s test for equality of variances in Table 9, F = 36.941 and p < 0.001, indicating a significant difference in variance between the two groups, thus violating the assumption of homogeneity of variances. Therefore, the result from "Equal variances not assumed" was used. The t-test revealed t = 14.350, df = 29.000, and p < 0.001, indicating a highly significant difference. Furthermore, the effect size results were Cohen’s d = 3.709 and η^2^ = 0.780, indicating an extremely large effect size for the observed difference. This confirms that the serious game developed based on AI and AR technologies significantly enhanced visitors’ knowledge acquisition of blue calico printing, and the intervention was significantly more effective than traditional museum visits.

**Table 8:** Enhanced value group statistics.

	Group	N	Mean	Std. deviation
Improvement Score	Experimental group	30	4.07	1.552
	Control group	30	0.00	0.000

**Table 9:** Enhanced value independent samples test.

		Levene’s test for equality of variances		t-test for Equality of Means				
		F	Sig	t	df	Sig. (2-tailed)	Cohen’s d	η^2^
Improvement score	Equal variances assumed	36.941	0.000	14.350	58	0.000	3.709	0.780
	Equal variances not assumed			14.350	29.000	0.000	3.709	0.780

#### User experience questionnaire analysis

The user experience questionnaire comprised 15 items, categorized into five dimensions based on their functional focus: Items 1 to 3 evaluated Fun, Items 4 to 6 assessed Immersion, Items 7 to 9 focused on Usability, Items 10 to 12 measured Interactivity, and Items 13 to 15 addressed Emotional Engagement.

Through descriptive statistical analysis using SPSS, it was found that the experimental group scored 3.16 on the Fun dimension, higher than the control group’s 3.04, indicating that the AI- and AR-based game content was more effective in stimulating user interest. The standard deviation for the experimental group was 1.49, slightly higher, suggesting a greater individual variance in experience ratings but generally good acceptance. For the Immersion dimension, the experimental group achieved an average score of 3.23, compared to 3.09 for the control group, suggesting that the integration of new technologies moderately enhanced user immersion. The similar standard deviations indicate relatively stable internal evaluations in both groups. In terms of Usability, the experimental group scored 3.58, compared to the control group’s 3.35, with a clear advantage, especially in aspects such as clear interface layout and intuitive operations. This suggests that the AI + AR system demonstrated sound design principles in terms of user interaction. The experimental group scored 3.57 on the Interactivity dimension, significantly higher than the control group’s 2.94. This highlights the success of the AI-driven virtual character “Master Wu” in enhancing user engagement and narrative immersion—one of the major strengths of the game design. Lastly, on Emotional Engagement, the experimental group scored 3.02, versus 2.77 for the control group, indicating that the AR-based contextual environment and cultural storytelling helped foster emotional resonance and a stronger willingness to recommend the experience.

**Fig. 8:**
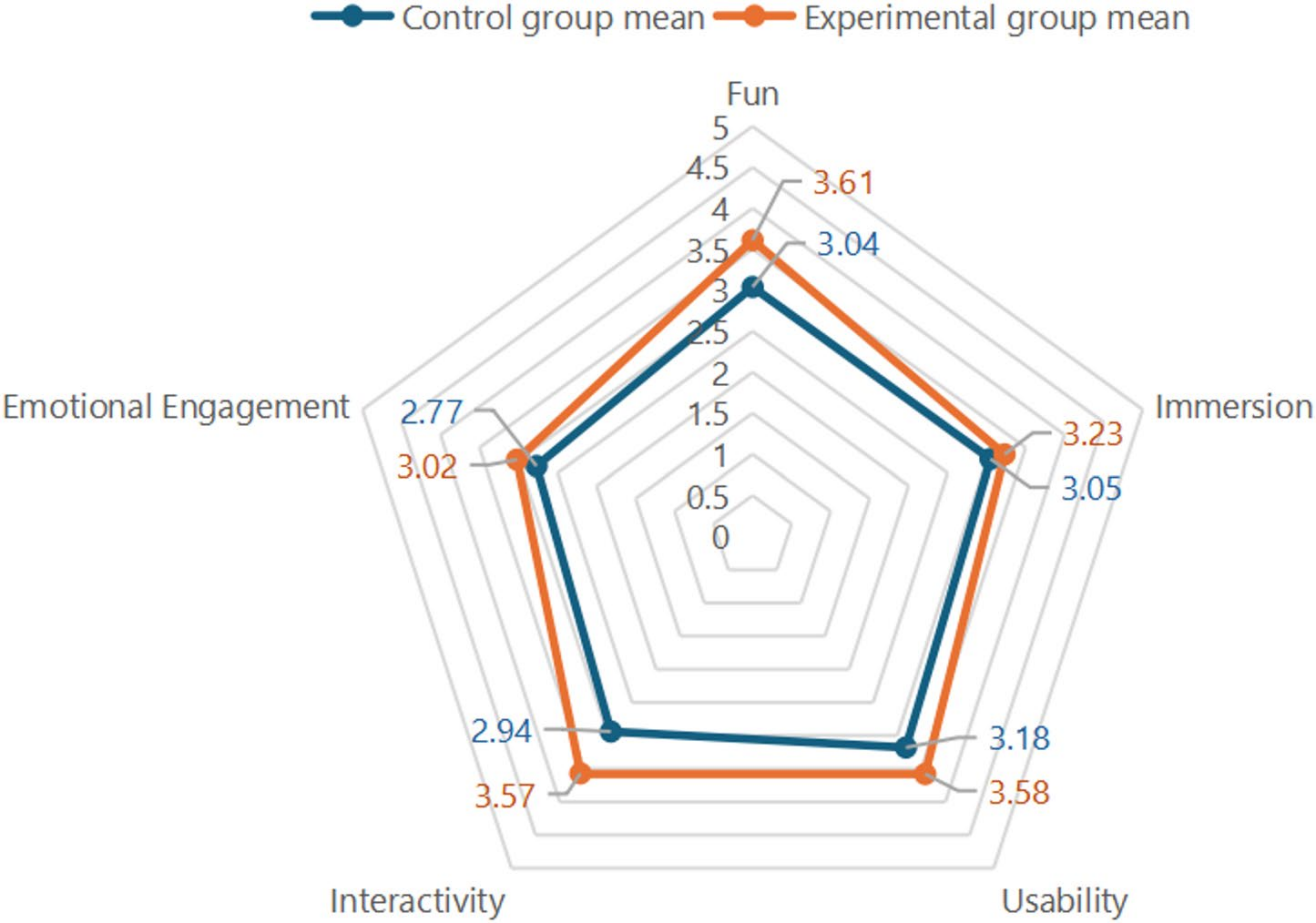
Comparison of scores between the experimental and control groups across five user experience dimensions.

To compare the score differences between the experimental and control groups across each dimension, a systematic statistical analysis was conducted, including independent-sample t-tests, effect size calculations, and mean score comparisons. The results of the independent-sample t-tests are presented in Table 10. The findings show that the experimental group outperformed the control group across all five user experience dimensions. The most prominent difference appeared in the interactivity dimension, where item 12 approached the significance level (*p* = 0.072), indicating that the AI + AR serious game demonstrated superior performance in enhancing user engagement and interactive experiences. Although other dimensions did not reach statistical significance, they all exhibited positive improvement trends, particularly in fun and usability, reflecting the potential value of technology-assisted design. A direct comparison of the average scores across the five user experience dimensions further refined these findings, as illustrated in Fig. 8. The experimental group performed significantly better in interactivity, fun, and usability, with differences exceeding 0.5 points in both interactivity and enjoyment. In contrast, immersion (3.23 vs. 3.09) and emotional engagement (3.02 vs. 2.77) did not show significant differences, though the experimental group still maintained a consistent advantage. Overall, the combined analyses indicate that the serious game developed in this study shows distinct advantages in promoting interactive participation and stimulating user interest, while further optimization is needed to deepen emotional resonance and immersive experience.

**Table 10:** Analysis of inter-group differences.

		Levene’s Test for equality of variances		t-test for equality of means		
		F	Sig	t	df	Sig. (2-tailed)
This game is very interesting	Equal variances assumed	0.527	0.471	-1.620	58	0.111
	Equal variances not assumed			-1.620	57.924	0.111
The quests and content in the game attracted me	Equal variances assumed	0.064	0.801	-1.688	58	0.097
	Equal variances not assumed			-1.688	57.923	0.097
The game process is enjoyable and not boring	Equal variances assumed	0.712	0.402	-1.222	58	0.227
	Equal variances not assumed			-1.222	57.553	0.227
The experience was very engaging, and time passed quickly	Equal variances assumed	2.735	0.104	-0.246	58	0.807
	Equal variances not assumed			-0.246	56.395	0.807
I was naturally immersed in the game’s story and mission situations	Equal variances assumed	1.669	0.201	-0.537	58	0.594
	Equal variances not assumed			-0.537	57.356	0.594
It creates an immersive feeling during the game	Equal variances assumed	0.855	0.359	-0.722	58	0.473
	Equal variances not assumed			-0.722	57.522	0.473
The system interface is clear and easy to operate	Equal variances assumed	0.316	0.576	-1.019	58	0.312
	Equal variances not assumed			-1.019	57.958	0.312
Able to successfully complete game tasks without the help of others	Equal variances assumed	0.000	1.000	-0.985	58	0.329
	Equal variances not assumed			-0.985	57.873	0.329
The system’s UI design makes me feel friendly and intuitive	Equal variances assumed	0.006	0.938	-1.442	58	0.155
	Equal variances not assumed			-1.442	57.812	0.155
Interacting with the AI ​​“Master Wu” made me feel like I was having a real conversation	Equal variances assumed	0.980	0.326	-1.692	58	0.096
	Equal variances not assumed			-1.692	57.262	0.096
The interactive approach during the game keeps me actively engaged	Equal variances assumed	2.805	0.099	-1.483	58	0.143
	Equal variances not assumed			-1.483	56.467	0.144
I can influence the course and content of the game based on my choices	Equal variances assumed	0.003	0.960	-1.833	58	0.072
	Equal variances not assumed			-1.833	57.998	0.072
I felt an emotional connection to the cultural content in the game	Equal variances assumed	0.340	0.562	-0.807	58	0.423
	Equal variances not assumed			-0.807	57.883	0.423
The game sparked my interest in learning more about indigo	Equal variances assumed	0.099	0.754	-0.558	58	0.579
	Equal variances not assumed			-0.558	58.000	0.579
I would recommend this experience to others	Equal variances assumed	0.696	0.407	-0.754	58	0.454
	Equal variances not assumed			-0.754	57.906	0.454

#### Impact analysis of the serious game on cultural communication effectiveness

The findings of this study indicate that serious games, as a task-oriented and narrative-driven communication medium, can effectively facilitate the learning and understanding of intangible cultural heritage. The comparative analysis of the pre-test and post-test results demonstrates that participants who engaged with the serious game exhibited significantly higher levels of knowledge acquisition regarding blue calico printing. This suggests that the serious game positively contributes to enhancing cultural knowledge retention.

In this game, participants are required to complete a series of tasks to unlock information about the history and craftsmanship of blue calico printing. Several interviewees mentioned that they unintentionally remembered a great deal of cultural knowledge while completing these tasks. This phenomenon suggests that the task-based mechanism of serious games can seamlessly integrate cultural content into the exploration process, thereby enhancing memory retention. Compared to traditional methods such as static exhibition boards or passive guided tours, this game-based approach encourages active engagement, increasing both visitor motivation and learning efficiency.

From the analysis of the user experience survey, it is evident that participants generally felt the game’s storyline and character design enhanced their sense of immersion, allowing them to stay focused and engaged throughout the experience. This immersive engagement further strengthened their sense of connection and identification with the cultural content, making the intangible heritage more tangible and personally relevant. Moreover, most participants expressed a willingness to recommend this form of experience to others, recognizing its combination of entertainment and educational value, and affirming its effectiveness in increasing both the acceptance and impact of cultural dissemination.

Therefore, serious games not only facilitate the understanding and acquisition of cultural knowledge on a cognitive level but also stimulate cultural identity and the willingness to share on an emotional level. This demonstrates their practical value and potential for broader application in intangible cultural heritage education and museum exhibition contexts.

## Discussion

### Exploring the extensibility of AI and AR technologies in the presentation of traditional culture

The serious game system developed in this study integrates AI and AR technologies, offering an innovative solution for the presentation and dissemination of blue calico, a form of intangible cultural heritage. Compared to traditional static exhibition models, this system overcomes the spatial and temporal limitations of conventional displays. By leveraging intelligent interaction and the fusion of virtual and physical elements, it opens a new pathway for the preservation and transmission of cultural heritage.

In the system design, an AI-powered virtual guide character named “Master Wu” was developed by integrating a large language model (LLM) interface. This design goes beyond the one-way information delivery of traditional audio guides, enabling human-like and context-aware two-way interaction.

User feedback indicated that conversations with the AI character “Master Wu” created a sense of authentic dialogue. Through role-playing, emotional warmth was infused into the cultural narrative, transforming users from passive recipients into active participants. Meanwhile, the AR-based image recognition and navigation functions intelligently link physical exhibits with digital content. When users scan specific patterns or tools, the system overlays illustrations, animations, and historical background information, enriching the presentation of cultural knowledge. During testing, this interactive mode was found to significantly alter visitor trajectories—most participants actively sought out marked exhibits to scan and explore, shifting their behavior from casual browsing to goal-oriented exploration, effectively reducing the likelihood of missing important artifacts. From a practical perspective, this flexible approach maintained the integrity of the visitor experience while avoiding costly equipment, making it particularly suitable for small and medium-sized museums by providing a lightweight, deployable, and cost-effective digital interaction model that enhances exhibition experiences.

From a broader applicability perspective, the proposed AI + AR integrated exhibition model is not limited to textile heritage such as Blue Calico, but also holds potential for a wide range of cultural heritage contexts—including ceramics, woodcarving, folk crafts, historical architecture, and traditional festivals. Such systems can be customized and extended according to the unique cultural characteristics of each domain: AI technology can handle knowledge explanation, contextual dialogue, and learning feedback, while AR overlays virtual information in real space, enabling an experiential mode of “seeing, feeling, and learning.” This model provides replicable and scalable digital solutions for small and medium-sized museums and local cultural exhibition spaces, improving the accessibility and dissemination of cultural content and promoting the sustainable development of traditional culture in education, tourism, and public communication.

Through the synergistic application of AI and AR, this system not only expands traditional exhibition models technologically but also deepens user engagement and understanding at the experiential level, offering a practical case for the digital dissemination of intangible cultural heritage.

### Limitations and recommendations

This empirical study found that such a museum visiting approach demonstrated positive effects on knowledge dissemination, interactivity, and enjoyment, but showed relative limitations in immersion and emotional engagement. The main reason for this discrepancy lies in the overly goal-oriented nature of the current game design, which lacks emotionally resonant narratives and fails to construct a deeply immersive contextual atmosphere. As a result, users tend to focus more on task completion rather than the depth of cultural experience.

To further understand the limited improvement in immersion and emotional engagement, future research should strengthen both qualitative and quantitative analyses of players’ behavioral data. In addition to the existing questionnaire data, behavioral indicators such as interaction frequency, task dwell time, and navigation paths can be recorded through the system backend. These data would enable a deeper analysis of players’ exploratory behaviors during gameplay and help determine whether the AI character effectively stimulates players’ exploratory motivation.

The application of AI and AR technologies in intangible cultural heritage (ICH) exhibitions also faces multiple challenges. From a technical perspective, accessibility and user acceptance remain limited. The current interactive interface is not sufficiently friendly for elderly visitors or those with limited digital literacy, as it requires a period of familiarization with the scanning and dialogue mechanisms. This can increase cognitive load during visits and disrupt the fluency of the immersive experience. At the content level, two key issues must be addressed: First, most current game modules are one-time experiences, leading to low willingness for repeated use and posing a challenge to content sustainability. Second, the authenticity of cultural representation demands careful consideration — AI-generated content may risk factual inaccuracies or excessive gamification. Achieving a balance between engagement and authenticity, while ensuring cultural narrative accuracy, is a crucial ethical concern for future AI-integrated design.

Furthermore, the system currently lacks social sharing and knowledge integration functions, which limits secondary cultural dissemination and prevents users from reviewing acquired knowledge after their visits.

To address these limitations, several improvement directions are proposed. First, by introducing a user profiling mechanism, the system can offer customized interaction modes for visitors of different ages, cultural backgrounds, and levels of digital proficiency, thereby lowering the usability barrier. Second, beyond task-oriented structures, the game mechanics should emphasize narrative enhancement, reinforcing storylines and contextual settings to evoke emotional resonance and a sense of immersion. To ensure sustainability and content authenticity, a content updating system and cultural review mechanism should be established. Finally, future designs may consider generating personalized learning records or digital game memorabilia, enabling visitors to consolidate and reflect on the cultural knowledge they have acquired.

### Recommendations for future museum-based cultural education

With the advancement of digital technologies, the educational role of museums should no longer be confined to static displays and information dissemination. Instead, it should evolve toward immersive, interactive, and personalized modes of communication. Based on the experience and data feedback from this study, the following recommendations are proposed for future cultural education in museums: First, the integration of AI and AR technologies should directly serve the core objectives of cultural education. These technologies should not merely function as tools for technological demonstration but rather enhance users’ initiative and engagement in the cultural learning process. Second, greater emphasis should be placed on the organic integration of gamification mechanisms with cultural content. Cultural transmission should not be a passive layer imposed on gameplay; instead, it should be embedded as the core of game tasks, narrative structures, and character design. Finally, hybrid models that combine online and offline cultural dissemination should be explored. Platforms such as Web AR, mini-programs, and virtual exhibition halls can facilitate the sustained, social, and networked transmission of cultural knowledge.

## Research limitations and future directions

This study conducted a systematic design and empirical analysis of the integration between serious games and intangible cultural heritage dissemination. However, several limitations remain in the research design and methodological implementation, which warrant further improvement in future studies.

Firstly, the study involved a relatively small sample size, comprising only 60 participants, primarily from the student demographic. This limited sample scope may affect the generalizability of the research findings. Future studies should consider expanding the sample size and including a broader range of user groups—such as family visitors, elderly participants, and cultural tourism audiences—to enhance the representativeness and applicability of the results.

Secondly, this study only evaluated short-term learning outcomes by comparing pre- and post-intervention test results conducted on the same day. As a result, the long-term retention of cultural knowledge acquired through the intervention was not examined. Considering that cultural heritage education often depends on cumulative and sustained learning processes, future research should incorporate delayed post-tests to assess the persistence of learning outcomes over time. This approach would enable researchers to determine whether digital game–based interventions can effectively support the long-term retention of cultural knowledge and contribute to the establishment of sustainable cultural heritage education models in higher education settings.

Thirdly, the same set of questions was used for both the pre-test and post-test, and although the question order was randomized to mitigate recall bias, participants’ familiarity with the test content may still have influenced their scores, potentially distorting the assessment of actual knowledge gains. Future research could consider employing question banks or alternative evaluation tools to enhance the validity of the measurement.

Furthermore, the current evaluation methods mainly focus on pre- and post-tests and user experience questionnaires. Although semi-structured interviews were later conducted and behavioural data during gameplay were collected, the study still lacks in-depth analysis of specific behavioural metrics such as interaction frequency, navigation paths, and time spent on tasks. This limitation constrains a deeper understanding of participants’ cognitive processes and learning mechanisms. Future research should consider implementing front-end tracking mechanisms within the game system to collect detailed behavioural data at different task nodes, thereby enabling a more nuanced exploration of how game mechanics influence cultural learning pathways. Therefore, upcoming studies could enhance research rigor and generalizability by diversifying participant samples, evaluating long-term learning outcomes, adopting multiple assessment tools, and analysing in-game behavioural data.

Meanwhile, future versions of the system could incorporate learning analytics mechanisms to record players’ decision paths, task completion times, and question–answer interactions during gameplay. These data could be visualized through personalized feedback dashboards, enabling students to conduct self-evaluation and plan their learning strategies, thereby strengthening the cycle of self-regulated learning. By reflecting learners’ behavioral trajectories and interest patterns, the system may help students better understand their learning progress while also providing references for future learning directions or career development. Such a data-driven feedback mechanism could further enhance the educational depth and long-term impact of serious games in cultural heritage education.

This study proposes a museum-based serious game tour model supported by AI and AR technologies, which demonstrates strong educational potential in the context of cultural dissemination. The personalized feedback enabled by AI, combined with the immersive interactive experiences afforded by AR, offers valuable insights into the application of serious games for intangible cultural heritage (ICH) education. In particular, the approach shows distinct advantages in enhancing cultural affinity and increasing the willingness to share and promote cultural knowledge. Future research should further explore the adaptability of this AI- and AR-supported serious game model in diverse cultural settings.

## Conclusion

This study developed the serious game “Dyeland” and explored its application in museums based on AI and AR technologies. The research findings indicate that visitors who engaged with the game demonstrated significantly better knowledge acquisition compared to those who did not use the game.

Regarding Research Question 1 raised in the introduction—How can AI and AR technologies enhance the interactivity and learning effectiveness of serious games in museums?—the study demonstrates that the integration of AI and AR significantly improves both aspects. The AI-driven virtual character “Master Wu,” equipped with natural language interaction capabilities, substantially increased the accessibility and engagement of cultural explanations. Compared to traditional audio guides, the frequency of user-initiated questions was notably higher, indicating greater interaction. Meanwhile, the use of AR image recognition technology effectively linked physical exhibits with in-game tasks, enhancing user immersion and reinforcing the educational impact of the experience.

Regarding Research Question 2—How can a balance be achieved between educational value and playability in museum-based serious games?—this study demonstrates that grounding game design in the core cultural elements of blue calico ensures that educational content remains central. Evaluation results show that the experimental group scored significantly higher than the control group in both educational and entertainment dimensions. This indicates that the game successfully integrated learning objectives with engaging gameplay, effectively achieving a balance between education and entertainment.

Regarding Research Question 3—How can the sustainability and long-term dissemination of cultural heritage be ensured in the design of serious games?—this study presents a lightweight, mobile-based solution tailored for the digital transformation of small to medium-sized museums. User experience survey results indicate that most visitors expressed a willingness to recommend the game, suggesting that this model possesses strong potential for sustained cultural dissemination. By combining accessibility, cultural depth, and interactive technology, the system supports ongoing engagement and promotes the long-term preservation and sharing of intangible heritage.

This study provides an innovative solution for the digital dissemination of traditional culture by integrating AI and AR technologies into a serious game format. On a theoretical level, the research addresses the common disconnect between technological application and cultural content in conventional digital museum studies. By transforming cultural elements such as blue calico patterns, production processes, and folk symbolism into interactive game mechanics, the study validates the effectiveness of gamification as a medium for cultural education. Furthermore, the research extends the Learning Mechanics–Game Mechanics (LM-GM) framework within a cultural context, achieving a balance between educational value and entertainment. In terms of assessment, a multidimensional evaluation system was constructed—encompassing both learning outcomes and user experience—offering methodological innovation for evaluating the effectiveness of serious games focused on traditional culture.

In terms of practical application, the lightweight technical solution developed in this study demonstrates significant potential for broader implementation. The mobile-based framework built on Unity and Vuforia substantially reduces technical barriers, enabling small and medium-sized museums to achieve digital transformation. By combining AI-driven semantic interaction with AR recognition technologies, the project facilitates a shift in museum interpretation models—from static content display to dynamic, context-rich storytelling. This approach not only enhances the accessibility and engagement of cultural knowledge but also offers a novel pathway for the dissemination of traditional culture in the digital era.

## Data Availability

The datasets used and/or analysed during the current study available from the corresponding author on reasonable request.
